# More Is not Always Better: The Differentiated Influence of Empathy on Different Magnitudes of Creativity

**DOI:** 10.5964/ejop.v14i1.1432

**Published:** 2018-03-12

**Authors:** Sven Form, Christian Kaernbach

**Affiliations:** a Institute for Psychology, Christian-Albrechts-University, Kiel, Germany; Department of Psychology, Webster University Geneva, Geneva, Switzerland; The Maria Grzegorzewska University, Warsaw, Poland

**Keywords:** empathy, everyday creativity, Big C Creativity, Little c Creativity, social interaction

## Abstract

Recently, researchers have argued about the importance of social aspects in creativity. Based on these arguments, one could hypothesize that if creativity is indeed about social aspects, then a social ability, such as empathy, will be relevant for creativity as an “interface” allowing the person to connect with others. A thorough review of the literature suggests that the association between empathic abilities and creativity may not be as straightforward as this hypothesis and also two recent empirical studies have suggested. This could be attributed to the fact that creativity may involve quite different levels such as creative achievement or everyday creativity. We suggest that social interaction, and with it empathy, plays a larger role in creative achievement than in everyday creative activities. Furthermore, we argue that too much empathy hinders everyday creativity. To explore the impact of empathy on different magnitudes of creativity, we applied two different self-report measures of creativity: creative achievement was measured by the Creative Achievement Questionnaire, while everyday creative activity was measured by the Creative Behavior Inventory. We used the Interpersonal Reactivity Index to measure empathy. Empathy had a positive correlation to achievement, but an inverted-U relationship to everyday creativity. We conclude that more connectedness is not always better for creativity. Therefore, the relevance of social aspects for creativity should not be generalized, but may depend on the magnitude of creativity considered.

There has recently been an intense debate among creativity researchers, whether social processes and aspects are relevant for creativity or not (Elisondo, 2016; Glăveanu, 2014; Gralewski, 2015; Lebuda, Galewska-Kustra, & Glăveanu, 2016; Runco, 2015), or—if one wants to avoid dichotomies (Glăveanu, 2015)—how much is creativity related to the influence of social aspects. The “how important” or “how much” raises the question whether the influence of social interactions on creativity can be quantified. A possible method to shed some light on this, in a quantitative way, is to look to what extent being connected or “tuned to” each other influences creativity: If social aspects and processes are important, as studies in eminent creative individuals suggest (Glăveanu et al., 2013; Simonton, 1984, 1992), a creative person will benefit from being connected to other people's minds and feelings. Expressed in quantitative terms, the better a person understands other’s minds and feelings, the better social aspects and processes could be included, and, thus, the more one's creativity should benefit from it.

On the other hand, there are also theoretical reasons why too much connectedness could hamper being creative. Creative individuals sometimes shield or withdraw themselves from the social environment (Feist, 1999). A high degree of connectedness is contradiction to the creative persons' need for autonomy. Autonomy, in turn, is important as it can facilitate creativity (Elsbach & Hargadon, 2006). The violation of social norms, commonly attributed to creative individuals, will be made more difficult, if one is concerned (or merely aware) that such behaviour might hurt other's feelings. In the most extreme cases, concern with the issues of other's may simply take away time from one’s own projects.

## Why Empathy?

The present study will explore the relevance of empathy, as it is a key factor to being connected to others. It can be defined as “the ability to understand and share in another's emotional state or context” (Cohen & Strayer, 1996). In this way, empathy connects the creative person to the individual worlds of other's. It is a multidimensional construct that not only includes affective, but also cognitive aspects, like the ability of taking someone else’s perspective (Davis, 1983). While there have been scattered empirical results, simply reporting a possible relation between empathy and creativity (see below), current research lacks theoretical reasons *why* specifically empathy should have a beneficial effect on creativity. The observed correlations could also be simply explained by the fact that empathy and creativity have a common neural basis as Takeuchi et al. (2014) discussed. For this reason, we offer some explanations, why empathy could be beneficial for creativity. While the first two mechanisms might be considered as improving the quality of a creative product, the latter mechanisms deal with the appreciation or the public perception of a product.

First, taking on another person’s perspective strengthens the association between intrinsic motivation and creativity, which leads to highly creative ideas (Grant, Berry, & Carolina, 2011). Given that perspective taking is a facet of empathy, this is a first hint that the overall construct could also be relevant for creativity.

Second, another aspect of empathy is the tendency to identify strongly with fictitious characters in books, films, or plays (Davis, 1980). Such a tendency should be useful in creating and depicting characters more authentically and thus making creative products more successful. The same might even apply to the depiction of characters in art work.

Third, empathy is a basic aspect of social skills (Riggio, Tucker, & Coffaro, 1989), probably because the ability to correctly receive and process interpersonal stimuli is essential for competent interpersonal functioning (reviewed in Morrison & Bellack, 1981). As such, empathy should be beneficial in social interaction, when persuading other's of the value of one's ideas and products (see also Kasof, 1995).

Finally, another application of empathy in the context of creativity might be derived from considerations by Simonton (2000):

[T]he creative individual must not produce work that is excessively original, for the product may then become incomprehensible, even to a degree that it provokes unpleasant emotional arousal (Berlyne, 1971). For example, empirical studies have found that the impact of an artistic product is often a curvilinear, inverted-U function of originality and similar aesthetic features (e.g. Kammann, 1966; Simonton, 1980, 1986, 1987; Steck & Machotka, 1975; Vitz, 1968). As a consequence, creators must somehow identify just the right amount of originality that will maximize success. (Simonton, 2000, p. 286)

Empathy could help the creator in anticipating the reaction of the social environment to one's creative product and adjust its features to “the right amount of originality”.

## Previous Empirical Studies

Although these theoretical considerations underline the benefits of empathic abilities, empirical studies, investigating the relation of empathy or closely related constructs to creativity, present an inconsistent picture. For example, creative students from copy-writing advertising courses differ not in their empathy to students of journalism (Auer, 1976). (It should be noted, however, that Auer defined and measured empathy not only with regard to understanding another individual, but also included the sensitivity to the social norm of a group. This is an aspect that is no longer included in more recent definitions of empathy [Davis, 1980, 1983]). Other studies investigated possible associations to creativity by using the TEIQue (Sánchez-Ruiz, Hernández-Torrano, Pérez-González, Batey, & Petrides, 2011; Sánchez-Ruiz, Pérez-González, Romo, & Matthews, 2015). The self-report measure TEIQue (Trait Emotional Intelligence Questionnaire) assesses, among other emotion related factors, *emotionality.* Emotionality is here of interest, as it incorporates the facets *empathy* and *emotion perception* (Petrides, 2009). While the term empathy is specifically used in the TEIQue for the capability of taking someone else’s perspective, emotion perception reflects clarity about other people’s feelings. The emotionality factor was neither significantly correlated with creative personality traits nor creative cognition (Sánchez-Ruiz et al., 2011, 2015).

Another study found similar results with no connection between constructs in question applying the EQ-i:YV with 5-17 year old students (Ferrando, 2006). The EQ-i:YV (BarOn emotional quotient inventory: Youth version) is designed to measure emotional intelligence in non-adults with an *interpersonal* dimension as one dimension among others (Bar-On & Parker, 2000). The test inventors defined this interpersonal dimension as understanding other people’s emotions, feelings and needs. There was no association between creative cognition and the interpersonal dimension (Ferrando, 2006).

While the studies mentioned so far did not find an association between creative and empathic abilities, other studies found a positive connection between both constructs. For example, a correlational analysis found a positive relation between past creative activities and empathy (Carlozzi, Bull, Eells, & Hurlburt, 1995). In this study, empathy was measured with the Affective Sensitivity Scale, a media-based, multiple-choice test focusing on the affective aspects of empathy (Kagan & Schneider, 1980; Werner, Kagan, & Schneider, 1977).

A Japanese study also found a correlation between empathizing and creative cognition (Takeuchi et al., 2014). Empathizing, defined as the ability or drive to identify another person’s thoughts and emotions, was measured with the Japanese version (Wakabayashi et al., 2007) of the empathizing quotient questionnaire (Baron-Cohen & Wheelwright, 2004). However, a critic might argue that the low correlation found (*r* = .17) was only significant due to the large sample size (*N* = 895).

Most recently, an even larger study (*N* = 1112) took great effort to explore the association between the Czech version of the empathizing quotient questionnaire and different aspects of self-reported creative behaviour (Dostál, Plháková, & Záškodná, 2017). Empathizing was especially correlated to intrapersonal and interpersonal creativity (*r* = .49), which was measured with items like “Being able to work through my personal problems in a healthy way” or “Helping other people cope with a difficult situation” (Kaufman, 2012). Other aspects of creative behaviour correlated with *r* = .19 or lower with empathizing (Dostál et al., 2017).

Although all the studies mentioned differ in the way that they label and measure the construct empathy, the core aspect is the same regarding the perception of other's feelings and minds. From the empirical results mentioned, the two most recent studies (Dostál et al., 2017; Takeuchi et al., 2014) seem to be able to give support in favour of a weak association between empathic abilities and creativity, simply due to their large sample size.

However, it is worth mentioning a meta-analysis that included about 4,000 individuals (Feist, 1998) as its results are also relevant here. A comparison of groups showed different results concerning characteristics related to empathy: Artists and scientists were more egocentric and hostile (and accordingly, presumably less empathic) than non-creative individuals. Furthermore, artists particularly demonstrated more coldness, which also leads to the assumption that they consider other's feelings and thoughts less. A similar conclusion can be derived from a more recent study, in which marketing students and artists scored lower than the control group in a subscale, consisting of friendliness and the concern for others (Martinsen, 2011).

## The Present Hypothesis

A possible solution for these seemingly contrasting results could lie in the different significance of the social environment for different magnitudes of creativity. On the one hand, there is Big C creativity, focusing on publicly acknowledged achievement, as creative work should be “accepted as tenable or useful or satisfying by a group” (Stein, 1953, p. 311). In contrast, little c creativity is possible in private without appreciation or social recognition (Runco, 1995, 2015). Such everyday creative activity is more about the personal experience during the process (Moran & John-Steiner, 2003). Accordingly, the different relevance of other people for the magnitudes of creativity is meant when speaking of “internal and external frames of reference” (Stein, 1953, p. 312). As the social environment has different importance, the ability of empathy as an “interface,” connecting environment and person, should have a different impact as well, depending on the magnitude considered.

What is seen as achievement needs to be communicated and is judged by the social environment, representing an external frame of reference. The higher one climbs the “achievement ladder”, the more social interactions probably occur and the more they become important (e.g. book signings for authors, contact with publishing agents).

Everyday creative behaviour certainly does not completely exclude social interactions. Thus, a certain level of empathy should be helpful for those interactions. However, everyday creativity is not subject of evaluation by others as much as achievement. If one wants to practice one’s creative activities in private, one can easily do so, representing an internal frame of reference. In fact, everyday creative activity is more associated with being creative independently from the social environment than achievement (Form, Schlichting, & Kaernbach, 2017). While a certain level of empathy is needed (or at least helpful) for social interactions arising from creative activity, too much empathy should be a drawback. For example, high empathy could make it difficult to address one's own conflicts or work through negative experiences, which are known benefits from everyday creativity (Richards, 2010). More generally, high empathy will marginalise the creative experience in the act of making. In other words, the focus should shift from an internal frame of reference to an external frame of reference. Thus, high empathy should do more harm than good for everyday creative activity.

We accordingly hypothesized an inverted-U relationship between empathy and everyday creative activity. In regard to creative achievement, we expect a positive linear relation with empathy, due to the stronger interaction and dependence on the environment.

## Material and Methods

### Participants and Satistical Analyses

Participants were recruited via notices displayed in public locations, via personal networks and by personally approaching individuals at the campus of the Muthesius Art Academy in Kiel. Thus, twenty percent of individuals were from the field of fine arts, communications design, industrial design, arts education or spatial design. The rest were students from the University of Kiel, mainly psychology undergraduates. The whole sample included 106 individuals (54.7% females). The mean age was 25.2 years (*SD* = 7.9 years, range = 18-58 years, *Mdn* = 22 years). All participants gave informed consent prior to participation.

Analyses were performed with SPSS Statistics 20.0 Software (IBM SPSS Inc. Chicago, IL). As creative achievement generally shows right-skewed distribution, we log-transformed the CAQ-scores for all statistical analyses as has been suggested earlier (Silvia, Wigert, Reiter-Palmon, & Kaufman, 2012).

### Instruments

In order to measure creative achievement, participants gave self-reports in the form of the German version (Form et al., 2017) of the Creative Achievement Questionnaire (CAQ, Carson, Peterson, & Higgins, 2005). The CAQ specifically asks for accomplishments in ten creative fields, so called domains such as “visual arts”, “creative writing” or “scientific discovery”. It emphasizes the “Big C-creativity” (Silvia et al., 2012) or in another terminology “Pro c-creativity” (Kaufman & Beghetto, 2009): highly creative contributions, accepted and acknowledged by the social environment. Example items are “I have had a showing of my work in a gallery.” or “I have sold one of my inventions to people I know.” The German version of the CAQ has good concurrent and discriminant validity (Form et al., 2017). Because items of the CAQ scale are not like Likert-items, but based on a step-wise model, we do not report Cronbach’s alpha for the CAQ (Silvia et al., 2012). See Table 1 for descriptive statistics of the present sample.

**Table 1:** Descriptive Statistics and Correlations

Scale	Min	Max	*M* (*SD*)	Skewness *(SD)*	Kurtosis *(SD)*	CBI	LogCAQ
CBI	7	167	63.3 (32.6)	0.7 (0.2)	-0.4 (0.5)		
LogCAQ	0	1.6	0.9 (0.3)	-0.6 (0.2)	-0.6 (0.5)	.45**	
SPF	21	47	42.3 (8.5)	-0.4 (0.2)	1.3 (0.5)	.19	.22*

*Note. N* = 106, CBI = Creative Behavior Inventory, logCAQ = log-transformed CAQ, SPF = sum of the three subscales of Saarbrücker Persönlichkeitsfragebogen (empathy).

*p < .05. **p < .01.

We measured creative activity with a self-translated German version of the Creative Behavior Inventory (CBI, Hocevar, 1979). The German version^i^ is a 74-item questionnaire asking about (extracurricular) creative activities and behaviours and how often they had occurred in the past. Participants rated items (“I painted an original picture” or “I designed and made my own greeting cards”) according to frequency, on a 5-point ordinal scale. Although CBI and CAQ-scores correlate, the correlation is low enough (*r* = .45, *p* < .01) to support the discriminant validity of both tests. Cronbach's α for the original CBI is in the range 0.63-0.89 (Hocevar, 1979, 1980). In our sample, Cronbach's α was .83. Scores were normally distributed (Shapiro-Wilk, *p* > .05).

We used the SPF (Saarbrücker Persönlichkeitsfragebogen, Paulus, 2009) to measure empathy. It is the newest revised German Version of the IRI (Interpersonal Reactivity Index, Davis, 1980). Participants give information on 16 items, using a 5-point Likert scale, about the frequency of certain feelings and behaviours. It was validated in 812 individuals, with satisfying reliability (α = 0.78). Cronbach's α was .83 in our sample. While Davis (1983) originally did not recommend a general overall score with summing up the four different scales measured in the test, the four scales are correlated (ranging from .21 to .48, Paulus, 2012). Accordingly, the scales (respectively their factors) cannot be seen as independent. Using structural equation modelling, Cliffordson (2001) found that adding three of the four subscales serves best as a measure of a general empathic disposition. A comparison of models later confirmed this approach for the German version to be the best fitting method (Paulus, 2012). Thus, we follow this procedure by adding up the scores of the subscales *perspective taking*, *fantasy* and *empathic concern* [the latter also labeled as *compassion* (Leiberg, Klimecki, & Singer, 2011) or *sympathy* (Hortensius, Schutter, & de Gelder, 2016)]. The resulting SPF scores were normally distributed in our sample (Shapiro Wilk, *p* > .05).

## Results

**Figure 1:**
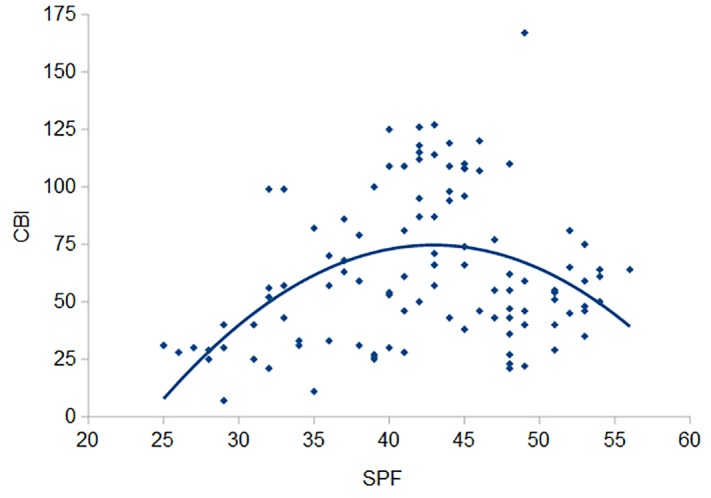
A scatterplot comparing self-reported empathy (SPF) and self-reported everyday creativity (CBI), which shows a curvilinear relationship (*N* = 106).

To determine whether empathy has a differentiated impact on varying levels of creative behaviour, we performed different regression analyses. For the first regression analysis, the empathy score was the independent and CBI the dependent variable representing the linear regression. Then, empathy was squared and added to the regression equation to test for curvilinearity. In the first model, empathy was a marginally significant predictor of everyday creativity (*p* = .052, β = 0.19, adjusted *R^2^* = .03). For the second model (adjusted *R^2^* = .19) the linear term (SPF: *p* < .001, β = 4.07) and the quadratic term were significant predictors (SPF^2^: *p* < .001, β = -3.90) (see Figure 1). Both the linear (SPF: *p* < .001, β = -3.72) and the quadratic term (SPF^2^: *p* < .001, β = -3.57) remained significant, when age (*p* > .05) and gender (*p* = .002, β = -0.27) were added as control variables.

Given that gender was a significant predictor of CBI and previous studies suggest significant gender differences for empathy (Eisenberg & Lennon, 1983; Schulte-Rüther, Markowitsch, Shah, Fink, & Piefke, 2008), we checked whether the quadratic relationship between SPF and CBI is moderated by gender^ii^. That is, does the extent of the bend of the function (its steepness or direction) depend on gender? The moderation analysis indicated neither the curvilinearity was more pronounced in one gender (SPF^2^ x Gender: *p* = .33, β = 1.23), nor did the direction depend on gender (SPF x Gender: *p* = .31, β = -1.32).

Finally, we examined the influence of empathy on creative achievement using another regression analysis. Empathy was a close to significant predictor of creative achievement (adjusted *R^2^* = .05, *p* = .057, β = .27). When SPF^2^ was included in the regression equation, the model did not improve.

## Discussion

The current study investigated whether empathy contributes in specific ways to different magnitudes of self-reported creative behaviour. In accordance with our hypotheses, empathy was associated with creative achievement in a linear fashion, while empathy had an inverted-U relationship with everyday creative activity. The effect even remained when controlling for age and gender, despite being female had also a positive effect on creative activity. The observation that achievement is linearly influenced by empathy, while creative activity is not, is reminiscent of Stein's (1987) argument that “we should not assume that the psychological characteristics associated with Creativity Little c are the same as those associated with Creativity Big C’’ (p. 420). Creative behaviour is possible in private (Runco, 1995, 2015), and thus involves less social interaction and is less dependent on judgement. The present results could be interpreted as taking this a step further: They suggest social interaction is not only less necessary, but a too pronounced connectedness to others can obviously be an obstacle, if one wants to focus on the experience in the creative act.

Our results are not necessarily in conflict with other studies, but add a more differentiated picture. While two studies found no association between creative cognition and empathic abilities (Ferrando, 2006; Sánchez-Ruiz et al., 2015), a larger Japanese study provided evidence for such an association (Takeuchi et al., 2014). This does not yet imply a relevance of empathy for creativity in general, but can also be explained by a common neural basis of both constructs as the authors argue themselves. In fact, when creativity was instead measured in terms of real-life creative activities, in an even larger study, empathy seemed to have a meaningful influence only on the specific aspect of interpersonal and intrapersonal creative behaviour (Dostál et al., 2017). For overall creativity, the effect of empathy was negligible, both in terms of creative activity and achievement. In regard to achievement, this result seems to contrast with the present result, which suggested an association with empathy. However, the contrast to the present study can be explained by differences in data transformation of the CAQ data. While Dostál et al. (2017) transformed raw scores into the ordinal categories low, medium, and high, we used a log-transformation as Silvia et al. (2012) suggested. An influence of empathy may have not been detected neither for overall (non-achievement) creative behaviors in Dostál et al.’s (2017) study, nor for studies using creative cognition tests (see earlier) due to a non-linear relationship.

A limitation of the present study is that we did not differentiate the effect of empathy for different domains, but a meaningful differentiation was not possible due to the present sample size. Another limitation is certainly the constrained focus on the creative person. If being connected to others is differently important for different magnitudes of creativity, then differences should be also found on the product and process level. For example, studies should ask whether there is a difference in experiencing the creative process between people, who show the same level of creative activity, but differ in their level of empathy. More precisely, are people with low empathy more concerned with their own feelings and thoughts and what happens to them in the act of making? Does someone high in empathy try to anticipate the reaction of audiences to one’s product? Investigating such questions would be opportunities to further tests the considerations from the introduction.

As Glăveanu (2014) recently criticized, creativity articles rarely offer practical implications or by being vague about them, we want to point out a possible consequence of our findings regarding the theory of creativity. While there probably is a consensus among creativity researchers that social aspects and processes have their influence, there is less consensus how far this influence goes. As an initial step to examine this issue, the present study suggests that the emphasis on the relevance of other's for creativity is justified as long as one talks about Big C Creativity, but that the topic could be more complicated for small c creativity and deserves more in-depth investigation.
